# Correspondence Between Effective Connections in the Stop-Signal Task and Microstructural Correlations

**DOI:** 10.3389/fnhum.2020.00279

**Published:** 2020-07-24

**Authors:** Fan Zhang, Sunao Iwaki

**Affiliations:** ^1^Graduate School of Comprehensive Human Sciences, University of Tsukuba, Tsukuba, Japan; ^2^Department of Information Technology and Human Factors, National Institute of Advanced Industrial Science and Technology (AIST), Tsukuba, Japan

**Keywords:** response inhibition, diffusion tensor tractography, probabilistic fiber tractography, hierarchical clustering, functional connectivity

## Abstract

Response inhibition is considered to involve the fronto-basal ganglia circuit including the inferior frontal gyrus (IFG), pre-supplementary motor area (preSMA)/SMA, subthalamic nucleus (STN), and the motor cortices, but it remains unclear whether there exists a correspondence between the anatomical and effective connections between these regions. We defined regions of interest (ROI) based on the results of our previous study, and subsequently used diffusion tensor imaging (DTI), especially probabilistic fiber tractography, for the identification of white matter tracts of interest. Accordingly, we extracted the fractional anisotropy (FA) from the tracts of interest and applied data-driven hierarchical clustering to examine whether a specific pattern exists in white matter tracts. We found three clusters in the fronto-basal ganglia circuits: (1) the IFG-SMA and IFG- STN; (2) the dorsolateral prefrontal cortex (DLPFC)-caudate and caudate-STN and caudate-IFG; and (3) the SMA-STN. Further investigation with pairwise linear inter-tract FA correlations revealed that there were significant correlations between specific pairs: (1) the DLPFC-caudate and caudate-IFG; (2) the caudate-IFG and IFG-SMA; (3) the IFG-SMA and SMA-STN; (4) the IFG-SMA and caudate-SMA; (5) the IFG-SMA and IFG-STN; (6) the SMA-STN and caudate-STN; (7) the SMA-STN and IFG-STN; and (8) the caudate-STN and IFG-STN. The combination of results from hierarchical clustering and microstructural correlations showed that probabilistic tractography infers effective connectivity: i.e., the DLPFC-caudate-IFG-SMA-STN pathway. Our results revealed that specific clusters in the fronto-basal ganglia circuit and certain pairs of white matter tracts with significant correlations predict the effective pathways (hyper-direct and indirect pathways) in response inhibition.

## Introduction

Response inhibition is the ability to voluntarily stop inappropriate actions when the environment changes. Successful behavioral control requires the involvement of two different forms of inhibitory processes, namely, proactive and reactive inhibition (Braver et al., 2007; Jaffard et al., 2007; Aron, 2011; Bari and Robbins, 2013; Mirabella, 2014). The former is goal-directed, preparing for restraining action before a stop signal (Chikazoe et al., 2009; Verbruggen and Logan, 2009), whereas the latter is triggered when a salient cue is observed and requires the complete abortion of action (Eagle et al., 2008a,b; Chambers et al., 2009; Aron et al., 2014). The prefrontal cortex (PFC), inferior frontal gyrus (IFG), supplementary motor area (SMA), the primary motor cortex (M1), and some basal ganglia, including the subthalamic nucleus (STN) and the striatum, have been shown to be part of the inhibitory network by various techniques such as functional magnetic resonance imaging (fMRI; Simmonds et al., 2008; Chikazoe et al., 2009; Chikazoe, 2010; Jahfari et al., 2010; Sharp et al., 2010; van Belle et al., 2014; Rae et al., 2015) and transcranial magnetic stimulation (TMS), transcranial direct current stimulation (tDCS), or deep brain stimulation with inhibitory control tasks (Coxon et al., 2006; van den Wildenberg et al., 2006; Chikazoe, 2010; Jahfari et al., 2010; Mirabella et al., 2012a,b, 2013; Cunillera et al., 2014; Rae et al., 2015; Duque et al., 2017; van Wouwe et al., 2017; Mancini et al., 2019). Furthermore, an electrocorticographic study including patients with pharmaco-resistant epilepsy revealed the causal involvement of the premotor area (PMA), primary cortex (M1), and Brodmann’s area (BA) 9 in a stop-signal task (Mattia et al., 2012). Also, a substantial proportion (30%) of monkey dorsal premotor cortex (PMd) cells produced signals predicting forthcoming actions in a reaching version of the stop-signal task, which suggests that both the M1 and PMd participated in the inhibitory control task (Coxon et al., 2006; Mirabella et al., 2011; Mattia et al., 2013). These areas combine with the basal ganglia to form a network that inhibits the activation of the M1 during reactive inhibition. Furthermore, converging evidence of functional interactions between these regions has suggested that response inhibition is realized through two pathways: the indirect (cortico-striato-pallido-subthalamo-nigral) and hyper-direct (cortico-subthalamo-pallidal) pathways (Alexander et al., 1986; DeLong, 1990; Mink, 1996; Aron et al., 2007a,b; Baker et al., 2010; Dunovan et al., 2015; Mallet et al., 2016; Zhang and Iwaki, 2019).

An open question in brain research is the relationship between the fMRI signals as a functional manifestation and the structure of the brain. Given that the neural underpinning for response inhibition has been studied using fMRI in the past decade, it is relevant to explore the linkage between anatomical connectivity and functional interactions (Werring et al., 1999; Honey et al., 2010). Diffusion tensor imaging (DTI) provides information regarding the structure of white matter by analyzing the degree of diffusion of water that is affected by local brain tissues (Basser and Pierpaoli, 1996; Pierpaoli et al., 1996). The cellular structure is assessed by the measurement of DTI parameters such as fractional anisotropy (FA), axial diffusivity (AD), and radial diffusivity (RD) in regions of interest as well as in tracts. In these parameters, FA reflects the degree of water anisotropy in local white matter (Basser and Pierpaoli, 1996; Pierpaoli et al., 1996); thus, the result characterizes the fiber density, axon diameter, fiber coherence, and myelination of white matter (Pfefferbaum and Sullivan, 2003; Büchel et al., 2004). RD links to the degree of myelination (Harsan et al., 2006; Tyszka et al., 2006), and AD indirectly reflects axonal degeneration (Harsan et al., 2006; Sun et al., 2006; Budde et al., 2009).

FA is a well-established parameter of microstructural organization and is widely used in white matter studies. Significant decreases in FA values are usually associated with diseases such as Alzheimer’s disease and Parkinson’s disease or physiological aging (Rose et al., 2000; Horsfield and Jones, 2002; Matsui et al., 2007; Hess, 2009; Westlye et al., 2010). A correlation has been revealed between such DTI parameters and behavioral performance (Klingberg et al., 2000; Niogi et al., 2008). It is generally hypothesized that the conduction velocity insides the nerves, which correlates with the reaction times, is determined by tissue microstructure (Fields, 2008; Seidl, 2014; Chevalier et al., 2015; Chopra et al., 2018).

Previous studies have investigated the relationship between the anatomical connections of the fronto-basal ganglia with response inhibition. Probabilistic tractography analysis between the basal ganglia and the IFG and preSMA/SMA has revealed significant correlations between the stop-signal reaction time (SSRT) and DTI parameters in Go/Nogo and stop-signal tasks (Liston et al., 2006; Casey et al., 2007; Rae et al., 2015). These studies have generally focused on the white matter tracts connecting the IFG, SMA, and STN and their correlation with the performance on cognitive tasks. The relationship between anatomical and functional connectivity has been well studied (Seghier et al., 2004; Riecker et al., 2007). Although the functional connections provide the temporal correlation between brain areas, they do not provide information on how these correlations are modulated; thus, it remains unclear whether a correspondence exists between the microstructural connections and the influence that one neural system exerts on another. The latter can be described as effective connectivity, which reflects the directed causal relationship between one brain area and another (Friston, 1994; Friston et al., 2003). Therefore, the investigation of the correspondence between white matter structure and effective connectivity in response inhibition will offer an opportunity to elucidate how functional and dynamic connections are generated and mediated directly or indirectly by anatomical structures.

In the present study, we selected tracts of interest based on the results of our previous study. We found significant activation of the IFG, SMA, dorsolateral prefrontal cortex (DLPFC), caudate, STN, and primary motor cortex (M1) in the stop-signal task, and further analysis of dynamic causal models revealed effective connections involving the aforementioned areas (Zhang and Iwaki, 2019). Then, we constructed the tracts of interest based on these regions: the DLPFC-caudate, caudate-IFG, IFG-SMA, SMA-STN, IFG-STN, and caudate-STN, and extracted the FA from the tracts of interest and applied hierarchical clustering to the white matter tracts. Furthermore, we examined the inter-tract correlations of FA with the pairwise linear correlation coefficient. These results were combined with results of a previous study revealing effective connectivity in response inhibition to investigate the correspondence between the structural and effective networks.

## Materials and Methods

### Stop-Signal Task and Participants

This study was approved by the institutional review board of the National Institute of Advanced Industrial Science and Technology (approval number: 2014-481). Eleven healthy right-handed adults (age range: 19–31 years; mean: 21.75 years; 8 male) with normal or corrected-to-normal vision were recruited from the University of Tsukuba as paid volunteers. All participants provided written informed consent before the experiment. Each participant was required to complete three runs of the stop-signal task paradigm in the scanner. Each run consisted of 40 “go” trials, 10 “stop” trials, and 10 “switch” trials.

**Figure 1:**
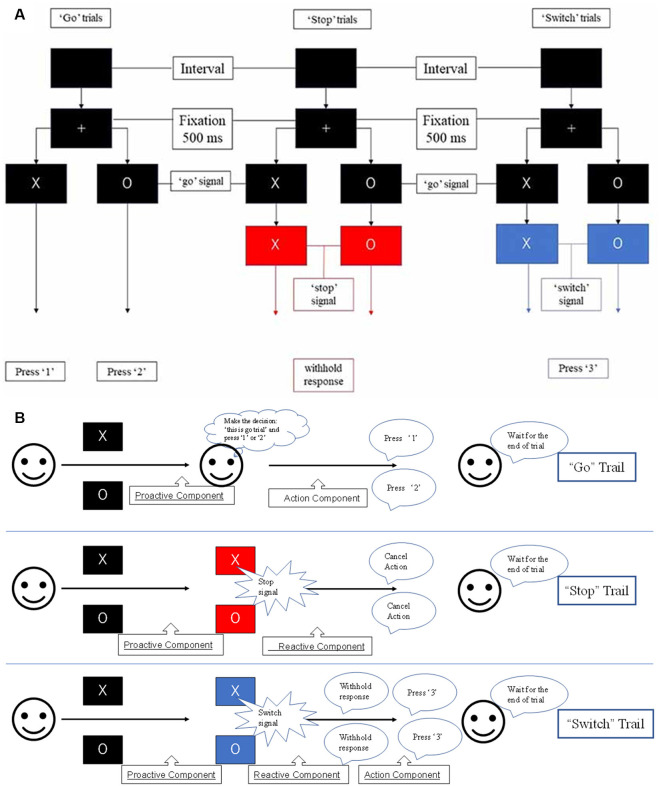
**(A)** Experimental paradigm used in this study. Participants were required to withhold their planned response and wait for any possible upcoming cue to avoid an incorrect response when the initial character (“X” or “O”) appeared. Participants had to entirely abort the responses that were already in progress if the background changed to red or switch their response to press “3” if the background changed to blue. **(B)** The stop-signal task consists of go, stop, and switch trials, which involve several cognitive components, i.e., proactive inhibition, reactive inhibition, or action. Based on this task design, reactive inhibition was analyzed by comparing the successful “switch” trials with successful “go” trials, while proactive inhibition was analyzed by the conjunction of all successful “go,” “stop,”’ and “switch” trials (Zhang and Iwaki, 2019).

At the beginning of each trial, participants watched a white fixation cross appearing on a black background for 500 ms; then, an “X” or “O” replaced the fixation cross for 1,500 ms. An equal distribution of the characters “X” and “O” was ensured across trials in random order. The participants had to press “1” if “X” appeared on the screen and press “2” if the character was “O.” The participants had to press the button as quickly as possible unless the color of the background changed after a while. If the color changed to blue, the participants were required to press “3” and entirely abort their planned response if the background color changed to red (Figure 1A; Zhang and Iwaki, 2019).

The stop-signal task consists of go, stop, and switch trials, which involve several cognitive components, i.e., proactive inhibition, reactive inhibition, or action (Figure 1B). When the initial character (“X” or “O”) appeared, participants were required to withhold their planned response and wait for any possible upcoming cue to avoid an incorrect response. Thus, all trials of the present study remained “uncertain” at the initial stage, i.e., participants were forced to adopt a proactive inhibition. An action component was involved when participants were presented with a “go” trial and pressed the corresponding button. For both “stop” and “switch” trials, participants needed to cancel the planned action that resulted in a reactive inhibitory component. However, in “switch” trials, participants had to press an alternative key, which led to a subsequent action component. Accordingly, the components in the “go” trials comprised a proactive inhibitory component and an action component (“proactive” + “action”). The “stop” trials were subdivided into a proactive and a reactive component (“proactive” + “reactive”), and the “switch” trials consisted of a proactive, a reactive, and an action component (“proactive” + “reactive” + “action”).

Based on this task design, reactive inhibition was analyzed by comparing the successful “switch” trials to successful “go” trials, while proactive inhibition was analyzed by the conjunction of all successful “go,” “stop,” and “switch” trials. Because fixed stop-signal delay (SSD) was used in the current procedure to improve the accuracy of participant behavior, we estimated the SSRT with the integration method (Logan and Cowan, 1984) by subtracting the SSD from the completion time that is determined by the distribution of no-signal go RTs.

### fMRI Data Acquisition

All fMRI scans were obtained using a 3-Tesla scanner (Ingenia 3T, Philips, Best, The Netherlands) at the Department of Information Technology and Human Factors, AIST (Tsukuba, Japan). Each participant’s head was fixed using foam padding to reduce head movement. Single-shot echoplanar imaging (EPI) sequences were used to acquire functional images. The EPI parameters were as follows: repetition time (TR) = 2,000 ms, echo time (TE) = 35 ms, flip angle = 90°, 31 ascending slices, thickness = 3.7 mm.

### DTI Acquisition

Imaging data were acquired on the same scanner. The diffusion images were acquired using a single shot echo-planar imaging sequence (TR = 18.486 ms, TE = 60 ms, flip angle = 90°, 32 gradient directions, matrix size: 224 × 224 × 140 mm (112 × 112 matrix), 2 mm slice thickness, 70 slices, b-factor = 1,000).

### Data Analysis

#### DTI Data Pre-processing

Pre-processing of the DTI data was performed using FSL 5.0 software (FMRIB’s Software Libary^1^) and Matlab 2015 (MathWorks, Natick, MA, USA). The B0 volume was first extracted and masked using “fslroi” and “bet.” Then, the diffusion images were corrected by “eddy_correct.” We further used “dtifit” to fit the corrected images and applied “bedpostx” to estimate the probabilistic tractography in each voxel.

#### Probabilistic Tractography Between the Frontal Cortex and Basal Ganglia

Group-level activations were found in the right IFG, left SMA, and bilateral STN in reactive inhibition, and brain regions with significant activations were identified as the DLPFC, caudate, IFG, SMA, and STN in both hemispheres in our previous study (Zhang and Iwaki, 2019). We expected that the IFG would be involved in both proactive and reactive inhibitory processes. Considering that the right IFG plays a critical role in response inhibition (see Aron et al., 2014; but see Swick et al., 2008; Federico and Mirabella, 2014; Mirabella et al., 2017; Mancini et al., 2019; Di Caprio et al., 2020 for a different view) and that no significant activation was found in the left IFG in reactive inhibition, we analyzed the inter-tract correlations of interest only in the right hemisphere. In our previous article, the dynamic causal modeling (DCM) analysis revealed that the M1 was not involved in either driving input (the regions in the model that first experience the neural changes caused by the manipulations of the experimental condition) or modulatory input (represents the specific experimental factor that modulates the intrinsic connections in the network). Furthermore, the M1 was considered to be the recipient of the frontal-basal ganglia-thalamic network command. Therefore, we excluded M1 from the analyses. Accordingly, we created masks of the right DLPFC, caudate, IFG, SMA, and STN in the Montreal Neurological Institute standard space; then, these prior masks were co-registered with the native space of each participant by the command “flirt.”

We used FSL to create the probabilistic tractography between the following seed regions of interest (ROIs): (1) the right DLPFC and right caudate; (2) the right caudate and right IFG; (3) the right IFG and right SMA; (4) the right SMA and right STN; (5) the right caudate and right STN; and (6) the right IFG and right STN. The probabilistic tractography was first estimated by applying FDT’s BEDPOST to build the distribution of diffusion parameters at each voxel with Markov Chain Monte Carlo Sampling (Behrens et al., 2003, 2007). Subsequently, FDT’s probtrackx was applied with 2,500 tract-following samples at each voxel in each seed area and terminated in the target regions. The whole tracts were built based on the probability distribution function, and only continuous tracts were retained. To extract the FA value of each tract, we first needed to transform these tracts in the MNI standard space into the individual space with the command “flirt.” Then, we created the probability maps for each subject by binarizing with a voxel by 0 or 1 by “fslmaths,” based on whether the streamline samples passed through the voxel or not. These individual binary maps were added and overlapped on each subject’s FA map, and we extracted the average time series by “fslmaths” and “fslstats.”

#### Hierarchical Clustering and Correlation Analysis

We applied hierarchical cluster analysis, an iterative approach that groups the most similar paths at each step, to build the hierarchy of clusters that group similar objects into groups. Hierarchical clustering modeling employed the functions in Matlab 2015. We used a dendrogram to visualize the specific patterns among white matter tracts with a type of tree structure. We considered six tracts: DLPFC-caudate, caudate-IFG, IFG-SMA, SMA-STN, IFG-STN, and caudate-STN, so that at the beginning of the basic process of hierarchical clustering, we would have six clusters, and each cluster would only contain one tract. We calculated the Euclidean distance between the pairs of FA of white matter tracts using the *pdist* function and located the closest pair of clusters. Then, we merged them into a new cluster using the linkage function. The distance was calculated again between the newly formed clusters and the original clusters. The above steps were repeated until a hierarchical tree was formed.

After the determination of clusters in white matter tracts, we measured the correlations between the tracts of interest by calculating the pairwise linear correlation coefficient. We used paired *t*-test to determine if there were statistically significant FA correlations between the regions (DLPFC-caudate, caudate-IFG, IFG-SMA, SMA-STN, caudate-STN, IFG-STN) with *p* < 0.05.

Lastly, we chose the connections that satisfy two conditions: (1) close distance in hierarchical cluster analysis (similarity physical property reflects the direct anatomical connection); and (2) significant correlation (influence on each other in the cognitive task). Thus, we first considered all possible anatomical connections between the tracts of interest and then excluded those tracts that do not satisfy the conditions described above.

## Results

### Behavioral Data and Group-Level Activation

We applied paired *t*-test on mean RTs for “go” and “switch” trials to test if there were significant differences between them. Significant differences (*t*_(10)_ = 6.47; *p* < 0.0001) were observed in mean correct reaction times between “go” (963 ms, range: 836–1,092 ms, SD: 74 ms) and “switch” trials (1,120 ms, range: 948– 1,350 ms, SD: 87 ms). The mean SSRT was 454 ms (range, 304–737 ms, SD: 96 ms). The mean 206 accuracies of “go” trials (0.890, SD: 0.117) were higher than that of ”switch" trials (0.853, SD: 0.165).

The results of our previous study (Zhang and Iwaki, 2019) revealed significant activations in the bilateral visual cortex, DLPFC, caudate, SMA, IFG, STN, and M1 by the conjunction of contrasts of all successful “go,” “stop,” and “switch” trials. Meanwhile, significant activations were found in the right IFG, left SMA, and left M1, as well as bilateral activation in the STN (Zhang and Iwaki, 2019). Subsequently, we calculated the pairwise linear correlation between the SSRT and FA values (Table 1) of the white matter tracts and did not find a significant correlation between them. Given that we did not find any correlation, we will not deal with behavioral data in the rest of the manuscript.

**Table 1:** FA values for white matter tracts.

	Sub1	Sub2	Sub3	Sub4	Sub5	Sub6	Sub7	Sub8	Sub9	Sub10	Sub11
DLPFC-caudate	0.2766	0.2965	0.3504	0.3500	0.3001	0.3371	0.3144	0.3212	0.3551	0.3910	0.3498
Caudate-IFG	0.2467	0.2951	0.3207	0.3180	0.2735	0.3188	0.3090	0.3261	0.3389	0.3746	0.2989
Caudate-STN	0.3166	0.2923	0.3201	0.3062	0.2823	0.3188	0.3407	03453	0.3519	0.3242	0.3463
IFG-SMA	0.3037	0.3135	0.3788	0.3291	0.3019	0.3597	0.3677	0.3696	0.4045	0.3473	0.3498
IFG-STN	0.3590	0.3138	0.3555	0.3291	0.3305	0.3576	0.3851	0.3435	0.3993	0.3823	0.3903
SMA-STN	0.3692	0.3592	0.4073	0.3895	0.3390	0.3808	0.3979	0.3746	0.4128	0.3774	0.3969

*FA, fractional anisotropy; DLPFC, dorsolateral prefrontal cortex; IFG, inferior frontal gyrus; SMA, supplementary motor area; STN, subthalamic nucleus*.

### Dynamic Causal Modeling

In our previous study, the optimal architecture of the model compared by Bayesian model selection with fixed-effect analysis revealed that the reactive modulatory input influenced the connections from the right IFG to the left SMA, while the proactive modulatory input modulated the connectivity from the left caudate to the right IFG. We also found that the “longer” DLPFC-caudate-IFG-SMA-STN-M1 pathway is attributed to proactive inhibition, whereas the “shorter” IFG-SMA-STN-M1 pathway is involved in reactive inhibition.

### DTI Data

**Figure 2:**
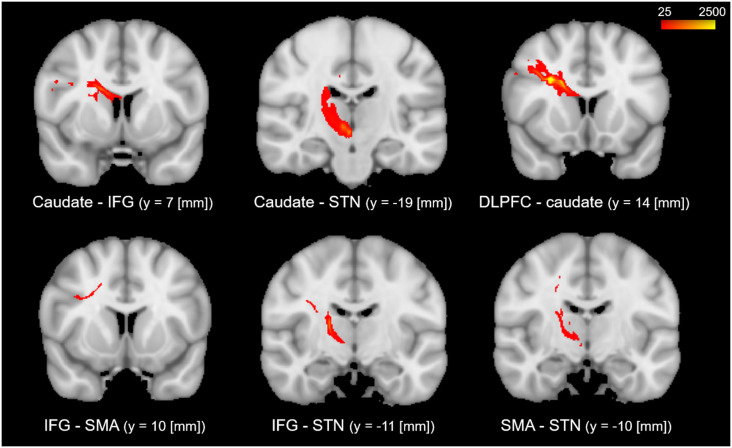
The probabilistic tractography between the regions of interest (ROIs). The color code denotes numbers of streamlines running through the voxels, which have maximum of 2,500. Voxels with value less than 25 have a probability (of being part of the tract) of less than 1%.

**Figure 3:**
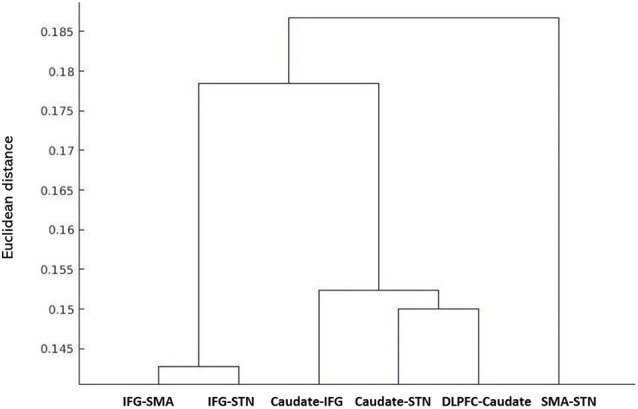
Hierarchical clustering of fractional anisotropy (FA) displayed as a dendrogram. The Y-axis denotes the Euclidean distance between the pairs of white matter tracts. SMA, supplementary motor area; STN, subthalamic nucleus; IFG, inferior frontal gyrus; DLPFC, dorsolateral prefrontal cortex.

The results of the probabilistic tractography are shown in Figure 2. Tractography was performed for the right hemisphere to delineate the tracts between each area in standard MNI space. In the resulting maps, voxels represented the probability of structural connections between given areas. All statistical maps were thresholded at a 1% probability of being part of the tract and registered to MNI standardized space. The similarity between the white matter tracts in the fronto-basal ganglia circuit displayed as a dendrogram showed that there were three significant clusters (Figure 3). The first linkage was between two pathways (IFG-SMA and IFG-STN), showing the most similarity compared to six association pathways. Further clustering analysis revealed a cluster including the DLPFC-caudate/caudate IFG/caudate-STN. The SMA-STN white matter tract was the most dissimilar compared to the other pathways.

**Figure 4:**
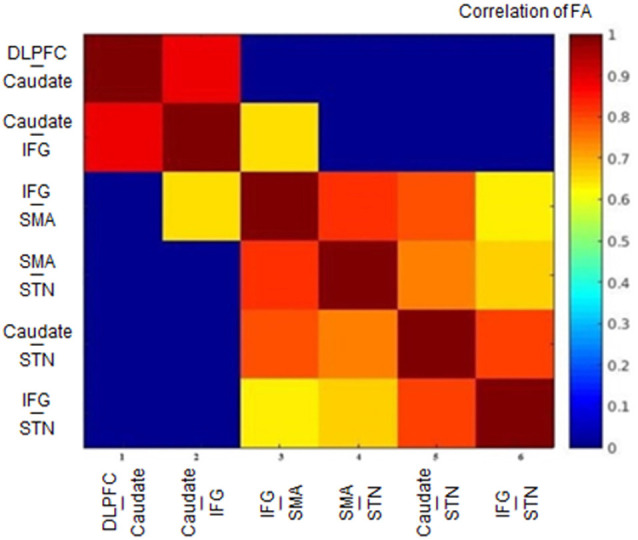
Heat map of the correlation matrix obtained from the tract-level FA. FA, fractional anisotropy; DLPFC, dorsolateral prefrontal cortex; IFG, inferior frontal gyrus; SMA, supplementary motor area; STN, subthalamic nucleus.

The FA correlations reflect the similarities of white matter tracts due to the ability to measure the degree of directionality of diffusion within a voxel (Conturo et al., 1999; Basser et al., 2000; Gossl et al., 2002). Thus, the results of the hierarchical clustering and the correlation matrix provided evidence of correspondence between the functional and structural pathways within the fronto-basal ganglia network, although some regions without direct structural connections exhibited strong functional connectivity. We then calculated the correlations between these tracts with the pairwise linear correlation coefficient (Figure 4; Table 2). Significant correlations were found between the following tracts (*p* < 0.05, DoF: 10): DLPFC-caudate/caudate-IFG (0.8843), caudate-IFG/IFG-SMA (0.6536), IFG-SMA/SMA-STN (0.8249), IFG-SMA/caudate-STN (0.7933), IFG-SMA/IFG-STN (0.6330), SMA-STN/caudate-STN (0.7481), SMA-STN/IFG-STN (0.6700), and caudate-STN/IFG-STN (0.8046).

**Table 2:** Matrix of pairwise linear correlation coefficients (and corresponding *p*-values) for FA between the white matter tracts of interest.

	DLPFC-Caudate	Caudate-IFG	IFG-SMA	SMA-STN	Caudate-STN	IFG-STN
DLPFC-Caudate	1	0.8843 (0.0003)	0.5703 (0.0670)	0.5598 (0.0733)	0.3972 (0.2265)	0.4866 (0.1290)
Caudate-IFG	0.8843 (0.0003)	1	0.6536 (0.0292)	0.4583 (0.1563)	0.4152 (0.2042)	0.3711 (0.2612)
IFG-SMA	0.5703 (0.0670)	0.6536 (0.0292)	1	0.8249 (0.0018)	0.7933 (0.0036)	0.6330 (0.0366)
SMA-STN	0.5598 (0.0733)	0.4583 (0.1563)	0.8249 (0.0018)	1	0.7481 (0.0081)	0.6700 (0.0241)
Caudate-STN	0.3972 (0.2265)	0.4152 (0.2041)	0.7933 (0.0036)	0.7481 (0.0081)	1	0.8046 (0.0028)
IFG-STN	0.4866 (0.1290)	0.3711 (0.2612)	0.6330 (0.0366)	0.6700 (0.0241)	0.8046 (0.0028)	1

*FA, fractional anisotropy; DLPFC, dorsolateral prefrontal cortex; IFG, inferior frontal gyrus; SMA, supplementary motor area; STN, subthalamic nucleus*.

## Discussion

In the present study, we investigated the correlation between white matter structure and effective connectivity in the same areas for response inhibition with a data-driven method. For the first time, we revealed specific clustering patterns as well as significant correlations between white matter tracts in the fronto-basal ganglia circuit for response inhibition. Furthermore, we found correspondence between the structural and effective pathways and provided evidence for the existence of hyper-direct and indirect pathways in anatomical networks.

### Specific Patterns of White Matter Tracts in the Fronto-Basal Ganglia Circuit

The dendrogram showed that strong homology existed in white matter connectivity as measured with FA between the DLPFC-caudate/caudate-IFG/caudate-STN, which is consistent with the results of previous studies. Comparative studies in monkeys and humans have revealed the anatomical connectivity between the DLPFC and the caudate (Albin et al., 1989; Parent, 1990; Parent and Hazrati, 1995; Lehéricy et al., 2004a,b) and the caudate is one of the main basal ganglion nuclei receiving axons from nearly the entire cortex (Maurice et al., 1998; Kolomiets et al., 2001, 2003). Furthermore, studies on anatomical and functional connectivity have proven that the caudate is one of the main input nuclei receiving inputs from the PFC and transferring information to the basal ganglia (Kunishio and Haber, 1994; Haber et al., 2000; Nakahara et al., 2002). A TMS study revealed that stimulation of the DLPFC increased neural activity in the caudate (Strafella et al., 2001; Knoch et al., 2006). The DLPFC-caudate circuit was also shown to be involved in proactive inhibition *via* the indirect pathway (Jahfari et al., 2011).

The homology between the IFG-SMA/IFG-STN is also supported by the results of previous studies. DTI and more advanced diffusion imaging methods have been used to study the connectivity of the SMA region in humans and have shown the connection between SMA and the fronto-opercular area (area 44 or “Broca’s area;” Lehéricy et al., 2004a,b; Klein et al., 2007; Oishi et al., 2008; Ford et al., 2010). There is converging evidence that the SMA and IFG play critical roles in controlling inappropriate response tendencies *via* their connections with the STN (Aron et al., 2007a; Jahfari et al., 2011; Rae et al., 2015). Research on inhibitory control has also provided evidence that the fronto-basal ganglia pathways support motor control *via* hyper-direct and indirect pathways. The former bypasses the striatum and directly connects the cortex and STN. The STN excites the GPi inhibiting the motor thalamic nuclei and the motor cortices. In the indirect pathway, cortical activity excites the striatum, which inhibits the GPe, releasing STN activity. STN, in turn, inhibits the motor cortices by decreasing motor thalamic nucleus activity (Aron and Poldrack, 2006; van den Wildenberg et al., 2006; Mirabella et al., 2012a, 2013; Mallet et al., 2016; van Wouwe et al., 2017; Mancini et al., 2019).

Studying the microstructural correlations among white matter tracts, we found that many, but not all of the strong homologous tracts, were tightly correlated. For example, we found that the white matter tracts DLPFC-caudate and caudate-IFG are strongly homologous in the hierarchical clustering analysis, and these tracts also revealed a significant correlation (0.8843, *p* < 0.05). However, although the white matter tracts DLPFC-caudate and caudate-STN also are strongly homologous, no significant correlation was found. Meanwhile, significant correlations were found in the caudate-STN and IFG-SMA tracts without homologous features. Thus, the presence of significant correlations between two tracts does not imply that they are homologous and *vice versa*.

### Inconsistencies Between Results From Hierarchical Clustering and Correlation Analysis of FA Data

The FA reflects the physical properties of fiber bundles such as packing density. In the hierarchical cluster analysis, we used Euclidean distance, which reflects anatomical similarity. Thus, closer distances represent more homologous tracts. The pairwise linear correlation coefficient reflects statistical correlations between tracts, which reflect the functional similarity between tracts. The statistical correlations between white matter tracts have been shown to reflect known patterns of phylogenetic development and functional specialization (Wahl et al., 2010; Li et al., 2012).

In the current study, we could confirm that most tracts with spatial overlap (DLPFC-caudate/caudate-IFG/caudate-STN that overlaps in the caudate; IFG-SMA/IFG-STN that overlaps in the IFG) were classified to the same cluster, denoting that the homologous tracts tended to cluster together. The dendrogram of hierarchical clustering showed that many, but not all, tracts that are in short Euclidean distance are in one cluster. The spatial overlap of tracts would influence their microstructure similarity. However, there were exceptions. For example, the IFG-SMA and caudate-IFG were found to overlap in the IFG, but the two tracts were classified into different groups. Furthermore, tracts DLPFC-caudate and caudate-STN were classified into one cluster with a short Euclidean distance but they were not statistically significantly correlated.

Thus, factors other than white matter microstructural connectivity such as FA also influence the functional correlation between tracts. Considering that the biophysical basis of white matter anisotropy is related to the effect of longitudinally oriented axonal membranes, the degree of myelination, and other factors, being physically homologous does not signify that the tracts are completely functionally correlated.

### Probabilistic Tractography Infers the Functional Pathway

**Figure 5:**
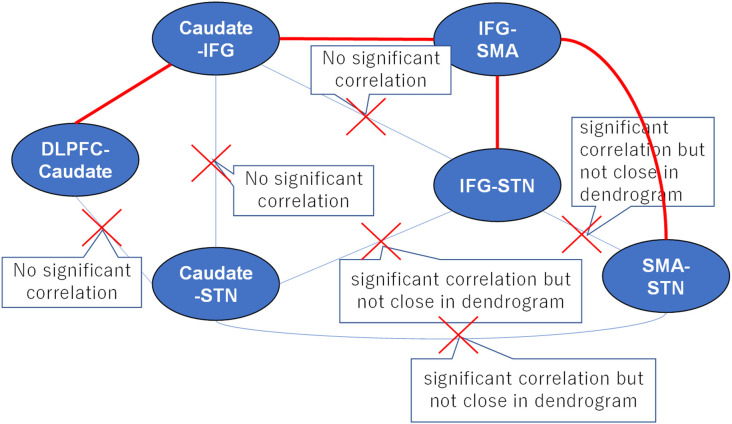
A proposed strategy of behavioral inhibition. The connections between tracts represent all possible anatomical pathways, and the red crosses denote pathways that were excluded because they could not simultaneously satisfy the requirements of hierarchical clustering and correlation analyses. Thus, the remaining red pathways represent the real anatomical pathways in fronto-basal ganglia circuits.

Numerous studies in humans and non-human primates have suggested that environmental cues are monitored by the DLPFC to adopt an adaptive motor strategy according to the environmental context (Watanabe, 1990, 1992; Asaad et al., 2000; Ragozzino, 2007; Hikosaka and Isoda, 2010). We selected the DLPFC-caudate as the main axis, and the associated tracts, i.e., the caudate-IFG and caudate-STN. We excluded the pathway between the DLPFC-caudate and caudate-STN because there was no significant correlation between the two tracts. For the same reason, we also excluded the pathways between the caudate-IFG and caudate-STN and caudate-IFG and IFG-STN. Although the pathways between the IFG-STN and caudate-STN were significantly correlated, the two tracts are in the same cluster. Thus, we excluded them. After this iterative method, the result revealed the anatomical pathways in the fronto-basal ganglia circuit (Figure 5). This result is consistent with the results of the DCM analysis in our previous article (Zhang and Iwaki, 2019), which revealed that the neural underpinning of proactive modulation is the effective connection from the DLPFC *via* the caudate to the IFG, while the subsequent effect of transmission is reflected in the effective connection of the IFG to the SMA in a common network. Thus, we found that the white matter tracts with significant correlations construct “effective” pathways: the DLPFC-caudate/caudate-IFG/IFG-SMA/SMA-STN pathway and the DLPFC-caudate/caudate-IFG/IFG-SMA/IFG-STN pathway.

In our previous study, we found that the “longer” pathway (DLPFC-caudate-IFG-SMA-STN) contributes to proactive inhibitory control, and a “shorter” pathway (IFG-SMA-STN) is modulated by reactive inhibitory control. In this study, the results of the clustering pattern and correlation analyses for the fronto-basal ganglia circuit were consistent with our previous results. Furthermore, the separate clusters revealed that the IFG-SMA white matter tract is less homologous compared with the DLPFC-caudate and caudate-IFG white matter tracts, which also supported the existence of two different pathways: the hyper-direct pathway and indirect pathway.

In this study, we did not find a significant correlation between FA and SSRT, which can be explained by the fact that task performance has a more complicated relationship with structural and effective networks. Our results from the hierarchical clustering and correlation matrix also suggested that there is a correspondence between the effective and structural pathways within the fronto-basal ganglia network, although some regions without direct structural connections exhibited strong effective connectivity. The divergence between the structural and effective networks may be attributed to the lack of correlation between structure and task performance. Also, different effective networks may share the same white matter tracts.

## Conclusion

The results of the hierarchical clustering and correlation analyses revealed that there is a correspondence between the structural and effective pathways in the fronto-basal ganglia circuit. Furthermore, we found that probabilistic tractography combined with statistical correlation can infer the effective pathway in response inhibition.

## Data Availability Statement

The datasets generated for this study are available on request to the corresponding author.

## Ethics Statement

The studies involving human participants were reviewed and approved by Institutional review board of the National Institute of Advanced Industrial Science and Technology. The patients/participants provided their written informed consent to participate in this study.

## Author Contributions

FZ and SI conceived and designed the experiments and planned and carried out the experiments. FZ performed the data analysis, interpreted the results, and wrote the manuscript with critical feedback from SI.

## Conflict of Interest

The authors declare that the research was conducted in the absence of any commercial or financial relationships that could be construed as a potential conflict of interest.

## Footnotes

^1^www.fmrib.ox.ac.uk/fsl
